# Engineering the immune response to "self" for effective cancer immunotherapy

**DOI:** 10.1186/2051-1426-2-S3-P22

**Published:** 2014-11-06

**Authors:** Shi Zhong, Karolina Malecek, Duane Moogk, Laura A Johnson, Zhiya Yu, Arsen Grigoryan, Eleazar Vega-Saenz de Miera, Farbod Darvishian, Wei Jun Gu, Katelyn McGary, Kevin Huang, Joshua Boyer, Emily Corse, Shao Yongzhao, Steven A Rosenberg, Nicholas P Restifo, Timothy Cardozo, Alan Frey, Iman Osman, Michelle Krogsgaard

**Affiliations:** 1 Xiangxue Pharmaceutical Co., Ltd, GuangZhou, Peoples Republic of China; 2 NYU School of Medicine, New York, NY, USA; 3 Perelman School of Medicine University of Pennsylvania, Philadelphia, PA, USA; 4 Surgery Branch, National Cancer Institute, National Institutes of Health, Bethesda, MD, USA; 5 New York University, New York, NY, USA; 6 North Shore-LIJ, New Hyde Park, NY, USA; 7 University of Minnesota, Minneapolis and St. Paul, Minnesota, MN, USA; 8 Roche, Zürich Switzerland; 9 US National Institutes of Health (NIH), Bethesda, MD, USA; 10 National Cancer Institute, Bethesda, MD, USA; 11 NYU Langone Medical Center, New York, NY, USA

## 

T cells play a critical role in host defense against viruses, intra- and extracellular microbes, and tumors. Because foreign antigen is presented amongst a vast majority of self-antigens, T cells have evolved the unique ability to discriminate "self" from "non-self" with high sensitivity and selectivity, enabling the elimination of foreign pathogens while largely avoiding self-reactivity. However, tissue-specific autoimmunity and tolerance to or eradication of cancer does not fit neatly into the self/non-self paradigm because the T cell responses in these situations are not directed to an exogenous pathogen, but rather most often to non-mutated self-proteins.

Therefore, an important question is how the immune system establishes suitable thresholds that allow positively selected T cells to interact with self-ligands in the periphery without causing overt activation. One hypothesis to explain how a T cell distinguishes among different types of self-ligands is the kinetic proof-reading theory, which relates signaling efficacy to the life-time of the TCR (T cell receptor)-pMHC (peptide-major histocompatibility complex) interaction. More recently, T cell maturation associated signaling feedback pathways have also been hypothesized to play a role in T cell discrimination of between self-ligands.

We are taking a variety of biophysical and cellular imaging approaches to determine how specific thresholds for T cell recognition of self-antigens are set. Our recent results [1] indicate that antitumor activity and autoimmunity are coupled and have a similar kinetic threshold; reducing autoimmunity cannot be accomplished without sacrificing efficacy of tumor killing. Therefore, an "optimal TCR affinity range" that leads to optimal tumor regression and minimal autoimmunity is elusive and treatment strategies focusing on increasing TCR affinities to a supraphysiological level has most likely little therapeutic benefit. Therefore, other approaches are needed to improve the balance between anti-tumor responses and autoimmunity.

Our strategy to overcome this issue includes novel methods for careful biophysical engineering of tumor-specific TCRs to carefully balance tumor-reactivity and autoimmunity. Furthermore, our recent preliminary data show that TCR-proximal signaling differs significantly between effector memory and central memory T cells due to differential constitutive activity and localization of signaling molecules. Understanding how activation signaling contributes to differences in memory T cell subset sensitivity may provide insight into how T cells can be manipulated to achieve optimal anti-tumor sensitivity. This could lead to adjuvants that target and enhance antigen-specific T cell anti-tumor efficacy. Together may lead to development of cancer immunotherapy approaches with improved outcomes.

Supported by NIH, The American Cancer Society, The Pew Trust and The Cancer Research Institute.
